# *Notch1* Mutation Represents a Potential Therapeutic Target to Enhance Immune Recognition in Oral Squamous Cell Carcinoma

**DOI:** 10.1002/cnr2.70345

**Published:** 2025-09-30

**Authors:** Takahiro Iwamoto, Kazuhiro Ogi, Takafumi Nakagaki, Takashi Sasaya, Sho Miyamoto, Koyo Nishiyama, Kenta Sasaki, Shintaro Sugita, Yasushi Sasaki, Akihiro Miyazaki

**Affiliations:** ^1^ Department of Oral Surgery Sapporo Medical University School of Medicine Sapporo Japan; ^2^ Department of Surgical Pathology Sapporo Medical University School of Medicine Sapporo Japan; ^3^ Biology Division, Department of Liberal Arts and Sciences, Center for Medical Education Sapporo Medical University Sapporo Japan

**Keywords:** CD8^+^ T cells, immunotherapy, *Notch1* mutation, oral squamous cell carcinoma, programmed cell death, tumor microenvironment

## Abstract

**Background:**

*Notch1*, a tumor suppressor gene, is one of the most frequently mutated genes in head and neck squamous cell carcinoma (HNSCC). Therefore, it is clinically important to investigate the effects of *Notch1* mutations on antitumor immunity in oral squamous cell carcinoma (OSCC), a subset of HNSCC.

**Aims:**

This study investigated *Notch1* mutations and the expression of immune‐related proteins. We also examined the influence of *Notch1* mutations on the immune microenvironment using a public database.

**Methods and Results:**

We examined the expression of *Notch1–4* in OSCC cell lines using qPCR. After *Notch1* knockdown, OSCC cell proliferation and migration were analyzed using CCK8 and wound healing assays, respectively. Localization of programmed cell death ligand 1 (PD‐L1) was assessed by western blot and flow cytometry, while PD‐L1 expression was evaluated by western blot. In the somatic mutation analysis of 47 OSCC patients, the relationship between tumor‐infiltrating CD8^+^ T cells and PD‐L1 expression was analyzed using immunohistochemical (IHC) staining. Furthermore, data from The Cancer Genome Atlas (TCGA) were analyzed using multiple online bioinformatics tools to compare the characteristics of *Notch1* mutations in HNSCC. The expression of *Notch1* varied depending on the OSCC cell line phenotype, and Notch1 mutation was significantly correlated with tumor growth but not with tumor infiltration. In *Notch1* knockdown, PD‐L1 expression on the tumor cell surface increased, while cytoplasmic PD‐L1 expression decreased. Among the 47 OSCC patients analyzed, seven (14%) had *Notch1* mutations. Of those, five patients (71%) exhibited high tumor‐infiltrating CD8^+^ T cells and *Notch1* mutation. Online bioinformatics analysis using the xCell algorithm revealed that Notch1‐mutated tumors had significantly higher levels of naïve CD8^+^ T cells compared to Notch1 wild‐type tumors.

**Conclusion:**

These findings highlight *Notch1* mutation as a potential therapeutic target for immune recognition in OSCC.

## 1 Introduction

Oral cancer is one of the predominant malignancies within the head and neck region. Current therapeutic modalities for oral cancer include surgical intervention, radiotherapy, chemotherapy, and targeted pharmacotherapy. The disease is characterized by high rates of recurrence and metastasis, resulting in a 5‐year survival rate of less than 50%. Additionally, there are currently no specific therapeutic targets for oral squamous cell carcinoma (OSCC) treatment. These challenges contribute to an overall 5‐year survival rate of approximately 50% in treated patients, highlighting the urgent need for improved treatment strategies. Immunotherapy has emerged as a promising approach for recurrent or metastatic OSCC; however, response rates remain modest (14%–18%), with a 6‐month progression‐free survival rate of 23% and a 1‐year survival rate of 36% [1, 2, 3].

The Notch family comprises four members, Notch1–Notch4, which are type I transmembrane receptors. Notch1 plays an important role in regulating the proliferation, differentiation, and apoptosis of diverse cell types in various organisms [4]. Upon ligand binding, the Notch1 intracellular domain (NICD) is cleaved from the cell membrane and translocated to the nucleus, where it binds to the transcription factor CSL (CBF1/RBPJκ) to activate the canonical Notch pathway. Recent sequencing studies have investigated head and neck squamous cell carcinoma (HNSCC), including OSCC, at various anatomical sites [5]. *Notch1* mutations are frequently detected in OSCC, alongside mutations in *TP53, CDKN2A, PIK3CA, and HRAS* [6]. *Notch1* mutations have also been observed near the ligand‐binding domain [7]. However, the role of the Notch signaling pathway in tumorigenesis remains debated and may differ depending on cell type [8].

A decade ago, it was reported that inhibiting the Notch signaling pathway downregulates programmed death 1 (PD‐1) expression in activated tumor‐infiltrating CD8^+^ T cells [9], suggesting a potential immunosuppressive role of Notch signaling in chronic viral infections and cancer. DAPT is a potent γ‐secretase inhibitor and indirectly inhibits the Notch pathway, as it is a substrate of γ‐secretase. For instance, blocking Notch signaling with a γ‐secretase inhibitor reduced tumor burden in a mouse model of HNSCC and was associated with decreased tumor levels of myeloid‐derived suppressor cells, tumor‐associated macrophages, regulatory T cells, and immune checkpoint molecules (PD‐1, CTLA‐4, T cell immunoglobulin and mucin‐domain containing‐3, and LAG3) [10]. Similarly, in a murine model of melanoma, reduced *Notch1* expression resulted in decreased infiltration of immunosuppressive cells and increased cytotoxic T cells [11]. Additionally, inhibiting Notch signaling promoted the cytotoxicity of tumor‐infiltrating CD8^+^ T cells and enhanced the production of pro‐inflammatory cytokines, including interferon‐gamma, tumor necrosis factor‐alpha, interleukin‐1 beta (IL‐1β), IL‐6, and IL‐8, by CD8^+^ T cells in patients with colorectal carcinoma. This was accompanied by reduced PD‐1 expression in tumor‐infiltrating CD8^+^ T cells, without influencing cellular proliferation [12].

Recently, it was reported that the relationship between Notch signaling and programmed cell death ligand 1 (PD‐L1) expression is significant in hepatocellular carcinoma [13]. PD‐L1 is primarily localized on the cell membrane, where it interacts with PD‐1 on T cells, enabling tumor cells to evade antitumor immunity [14]. However, the role of nuclear PD‐L1 remains unclear [15]. High PD‐L1 expression in tumors has been identified as a biomarker for improved sensitivity to PD‐1/PD‐L1 blockade therapies [16, 17]. Although anti‐PD‐1 and anti‐PD‐L1 therapies have demonstrated antitumor effects in clinical practice, their low response rates in some patients make complete tumor eradication challenging.

In our previous study, a potential relationship was found between mutations in the Notch signaling pathway, tumor‐infiltrating CD8^+^ T cells, and PD‐L1 expression, suggesting enhanced antitumor immunity that could benefit patients with recurrent or metastatic OSCC receiving immune checkpoint inhibitor (ICI) therapy [18]. We concluded that patients with Notch pathway mutations often exhibit abnormalities in other oncogenic or tumor suppressor genes, reflecting a higher tumor mutational burden that may serve as a target for CD8^+^ T cells. These cells recognize tumor‐derived neoantigens and improve the response to ICB therapy in patients with OSCC. Based on these observations, we recently hypothesized that the interplay between *Notch1* mutation and PD‐L1 expression could reprogram the tumor microenvironment (TME) and influence CD8^+^ T cell infiltration.

In this study, we analyzed Notch1 protein expression in normal oral fibroblasts and OSCC cell lines in vitro. To elucidate the role of Notch1 in OSCC, we conducted cell counting kit‐8 (CCK‐8) and wound healing assays. The relationship between aberrant Notch1 expression and PD‐L1 expression in OSCC cell lines was examined using a Notch signaling inhibitor or *Notch1* knockdown by western blotting (WB). Additionally, PD‐L1‐related biological behavior in OSCC cell lines with aberrant Notch1 expression was analyzed by fluorescence‐activated cell sorting (FACS). The present study aimed to investigate the relationship between *Notch1* mutations and PD‐L1 expression in patients with OSCC through CD8^+^ T cells immunohistochemical (IHC) staining and in patients with HNSCC using multiple online bioinformatics tools. This study focused on the tumor immunology associated with *Notch1* mutations in OSCC. Our findings suggest that the relationship between *Notch1* mutations and CD8^+^ T cells infiltration, based on large‐scale databases, may provide insights into potential therapeutic strategies targeting this interaction in HNSCC.

## 2 Methods

### 2.1 Cell Lines and Human Tissues Samples

The fibroblast cell line from the lip (KD) and human OSCC cell lines (HSC‐2, HSC‐3, HSC‐4, OSC‐19, OSC‐20, OSC‐30, OSC‐70, SAS, SAT, HO‐1‐u‐1, and KOSC‐3) were obtained from the Japanese Collection of Research Bioresources Cell Bank (JCRB, Osaka, Japan). Additional human OSCC cell lines (HOC‐119, HOC‐621, MOT, and SCC‐25) were kindly provided by Prof. Nobuyuki Kamata (University of Tokushima, Japan). All cell lines were authenticated using short tandem repeat (STR) analysis and confirmed to be mycoplasma‐free through routine quality control. All the cell lines were cultured in Dulbecco's modified Eagle's medium (DMEM; Invitrogen, Carlsbad, CA, USA) supplemented with 10% (w/v) fetal bovine serum (FBS; Gibco, Waltham, MA, USA) and 1% penicillin/streptomycin (P/S; Gibco). These cell lines were incubated at 37°C with 5% CO_2_.

Tumor tissue specimens from the 47 patients with OSCC were obtained from Sapporo Medical University as part of a previous study. All tissue samples were collected after obtaining written informed consent from patients before surgery, and OSCC was histologically confirmed by pathologists. This study was approved by the Ethical Review Committee of Sapporo Medical University (342‐3416). IHC staining of archival specimens was performed. OSCC stage and grade was not considered in the present study.

### 2.2 RNA Extraction, cDNA Synthesis, and Quantitative Real‐Time Polymerase Chain Reaction (qPCR) Analysis

Each cell line was seeded at 70%–80% confluence in 10 cm dishes. Diluted cell suspensions (5.0 × 10^5^ cells/mL) were then seeded into 6‐well plates and incubated overnight. Subsequently, total RNA was extracted from each cell line using an RNeasy Mini Kit (QIAGEN, Tokyo, Japan) according to the manufacturer's protocol. The cDNA was prepared using the High‐Capacity cDNA Reverse Transcription Kit (Thermo Fisher Scientific Inc., Waltham, MA, USA). qPCR was performed with a TaqMan Array 96‐Well FAST Plate Human Notch Signaling panel (Cat. #4418942 Thermo Fisher Scientific Inc.). The following TaqMan assays were used for human genes: *NOTCH1* (Hs01062011_m1), *NOTCH2* (Hs01050719_m1), *NOTCH3* (Hs01128541_m1), and *NOTCH4* (Hs00965889_m1). mRNA expression was normalized to *GAPDH* (Hs99999905_m1) as a control. The cycling conditions were as follows: 50°C for 20 s, followed by 40 cycles of 95°C for 10 s and 60°C for 20 s. The 2^−ΔΔCT^ method was used for the relative quantification of gene expression. Data were analyzed using a Real‐Time PCR Detection System (StepOnePlus; Applied Biosystems, Tokyo, Japan). For each sample, the transcript levels of technical triplicates were averaged and used for subsequent analyses.

### 2.3 Cell Proliferation Assay

OSC‐20, HSC‐3, HO‐1‐u‐1, and SAS cells (5.0 × 10^3^) were seeded into 96‐well plates in 100 μL of DMEM containing 10% FBS at 37°C with 5% CO_2_ for 24 h. After incubation, each well was treated with 0.4 μL of DAPT (10 mol/L) in DMSO, 10 μL of Notch1 siRNA (1 pmol) in Opti‐MEM with RNAiMAX, or medium alone (Mock). After 0, 24, 48, and 72 h of incubation, 10 μL of CCK‐8 reagent (Fujifilm Wako Junyaku Inc., Osaka, Japan) was added. After 2 h of incubation, the absorbance of each well was recorded at 450 nm using a MULTISKAN FC device (Thermo Fisher Scientific).

### 2.4 Wound Healing Assay

OSC‐20, HSC‐3, HO‐1‐u‐1, and SAS cells (1.0 × 10^5^) were cultured in a six‐well plate until a single layer formed, and the bottom of the plate was scratched with the tip of a 20‐μL pipette to form a wound. Afterwards, the cells were washed with phosphate‐buffered saline (PBS) (−) to remove unattached cells, and 2000 μL of DMEM containing 10% FBS was added. For the DAPT‐activated group, 0.4 μL of DAPT was added at the same time. After 0, 6, and 12 h of incubation in 5% CO_2_ at 37°C, cell migration was recorded by time‐lapse photography (WSL‐1800 CytoWatcher, ImageSaverT v.2.0.1; ATTO, Tokyo, Japan).

### 2.5 Whole‐Exome Sequencing and Variant Calling

Cell lines with disordered Notch1 expression were subjected to genomic analysis. One hundred nanograms of DNA were sheared to approximately 200 base pairs (bp) by sonication. The genomic regions of whole exons were captured by microarrays using synthesized oligonucleotide probes hybridized to fragmented genomic DNA samples. The libraries were sequenced using an Illumina NovaSeq 6000 with 2 × 150 bp paired‐end reads. Sequence exome enrichment was performed using SureSelect Human All Exon V6 kits according to the manufacturer's instructions and compared to the human reference genome b37 using an Agilent SureSelect Human All Exon V6 Kit (Agilent, USA). Briefly, raw sequencing data in FASTQ format were aligned against the reference human genome (b37) using SAMtools. The Genome Analysis Toolkit and Sentieon Genomics Tools were used for germline single‐nucleotide variants (SNV) and indel calling, and Sentieon Genomics Tools were used for somatic SNV and small indel calling. ANNOVAR was used to functionally annotate the genetic variants.

### 2.6 DAPT Administration and Notch1 siRNA Transfection

To determine the optimal concentration of DAPT (cat. #ab120633, γ‐secretase inhibitor; Abcam Plc., Cambridge, GBR), cells were seeded in 96‐well plates, and DAPT was added in stepwise dilutions starting at 20 μM. A final concentration of 10 μM DAPT was found to be the most effective. After 24 h of culture at 37°C, inhibition efficiency was assessed by WB, and this concentration was used for subsequent experiments. Next, cells were seeded in six‐well low‐attachment plates (1 × 10^3^ cells/cm^2^) and transfected with 25 nmol/L of control siRNA, Notch1 siRNA (Cat. ID: D‐001810‐10‐05, Dharmacon; Horizon Discovery Ltd.), or non‐targeting siRNA (Cat. ID: L‐007771‐00‐0005, Dharmacon) using Lipofectamine RNAiMAX Reagent (Invitrogen; Thermo Fisher Scientific Inc.) according to the manufacturer's protocol. For transient silencing of *Notch1*, the following four small interfering RNA (siRNA) sequences were used: 5′‐GCGACAAGGUGUUGACGUU‐3′, 5′‐GAUGCGAGAUCGACGUCAA‐3′, 5′‐GGACAUCACGGAUCAUAUG‐3′, and 5′‐GAACGGGGCUAACAAAGAU‐3′. For non‐targeting siRNA, the following four siRNA sequences were used: 5′‐UGGUUUACAUGUCGACUAA‐3′, 5′‐UGGUUUACAUGUUGUGUGA‐3′, 5′‐UGGUUUACAUGUUUUCUGA‐3′, and 5′‐UGGUUUACAUGUUUUCCUA‐3′. Cells were harvested 48 h post‐transfection for western blot analysis.

### 2.7 Protein Extraction and Western Blot Analysis

Total protein was extracted using RIPA buffer (Fujifilm Wako Junyaku Inc.) and a cell scraper on ice. The Pierce BCA Protein Assay Kit (Thermo Fisher Scientific Inc.) was used to determine the concentration of proteins. Each lane of 4%–12% Bis‐Tris gels (Thermo Fisher Scientific Inc.) was loaded with 30 μg of protein, which was transferred onto 0.22‐μm polyvinylidene difluoride (PVDF) membranes (2322453, ATTO) after electrophoresis. The PVDF membranes were blocked with 5% skim milk/PBS containing 0.01% Tween‐20 (PBST) for 1 h at room temperature and then incubated overnight at 4°C with the primary antibodies. The primary antibodies used in this study were Notch1 (1:1000, Cell Signaling Technology Inc., Danvers, MA, USA), cleaved Notch1 (1:1000, Cell Signaling Technology), Hes1 (1:1000, Cell Signaling Technology), PD‐L1 (1:1000, Cell Signaling Technology), and β‐actin (1:10000, Cell Signaling Technology) with gentle shaking overnight. After three washes with PBST for 10 min each, the membranes were incubated with horseradish peroxidase‐conjugated sheep anti‐mouse IgG (1:10000) or goat anti‐rabbit IgG secondary antibodies (1:1000) for 1 h at room temperature. After incubation and three washes with PBST for 10 min each, protein bands were detected using ECL Prime Peroxide Solution (Cytiva, Marlborough, MA, USA). Protein bands were visualized using an iBright 1500 imaging system (Invitrogen; Thermo Fisher Scientific).

### 2.8 Flow Cytometry Analysis

The cells were collected by trypsinization, followed by centrifugation at 500 × *g* for 5 min. Cell pellets were then resuspended in 100 μL of PBS (Life Technologies, Carlsbad, CA, USA). The following antibodies were used for FACS analysis: PE Mouse IgG1 (BioLegend, San Diego, CA, USA), PE‐anti‐human CD274 (B7‐H1, PD‐L1, BioLegend), and PE Mouse IgG1 κ isotype control (negative control; BioLegend). After incubating the cells with 1 μL of antibody for 20 min and washing with PBS, the cells were incubated at 4°C in the dark for 1 h. Cells were analyzed using a BD FACS CANTO II flow cytometer (BD Biosciences, San Jose, CA, USA). A minimum of 10 000 monocyte events were recorded for each sample based on a gate created on a scatter plot.

### 2.9 Evaluation of Immunohistochemical (IHC) Staining of PD‐L1 and Tumor‐Infiltrating CD8 ^+^ T Cells

A total of 47 samples were suitable for assessing PD‐L1 expression. PD‐L1 expression was assessed using PD‐L1 IHC (E1L3N XP Rabbit mAb #13684, Cell Signaling, 1:200) according to the manufacturer's instructions in paraffin‐embedded tumor samples obtained before treatment. Three expert pathologists from our institution assessed the expression of PD‐L1 based on the PD‐L1 combined positive score (CPS). CPS was scored as CPS ≤ 1%, 1% < CPS < 20%, and CPS ≥ 20%, based on the percentage of positively stained tumor cells in the sample.

For the CD8 IHC test, 12 paraffin‐embedded tumor samples with PD‐L1 expression (CPS ≥ 1%) were assessed using an anti‐CD8 alpha antibody (C8/144B, ab17147, Abcam) according to the manufacturer's instructions. Secondary antibodies were incubated with a DAB detection kit (DAB; Abcam, Waltham, MA, USA). The samples were then counterstained with hematoxylin (Dako, Carpinteria, CA, USA). CD8^+^ T cell density was assessed simultaneously using the DP2‐BSW software for an Olympus Microscope Digital Camera (Olympus Co., Tokyo, Japan) by three of the authors (TS, KS, and SS). To assess tumor infiltration and CD8^+^ T cell density in each compartment, at least three random fields were viewed, and in cases of heterogeneity, the value most representative of the entire section was used. The average number of CD8^+^ T cells used to stratify patients was 200. Then, we divided the count of CD8^+^ T cells into two categories: < 200 and > 200.

### 2.10 Datasets of Notch1‐Associated Immune Infiltration

TIMER 2.0 (https://compbio.cn/timer2/) was used to analyze the correlation between CD8^+^ immune infiltrates and Notch1 using TCGA datasets.

### 2.11 Statistical Analyses

All assays were performed in triplicate, and the data are presented as the mean ± standard error of the mean. Differences in test variables between the control and experimental groups were analyzed using either the Chi‐squared test (χ^2^ test) or Student's *t*‐test. Correlations between wild‐type and mutated *Notch1* were analyzed using Wilcoxon and Spearman rank correlations. Statistical significance was set at *p* < 0.05.

## 3 Results

### 3.1 Protein Analysis of *Notch1* in OSCC Cell Lines

**FIGURE 1:**
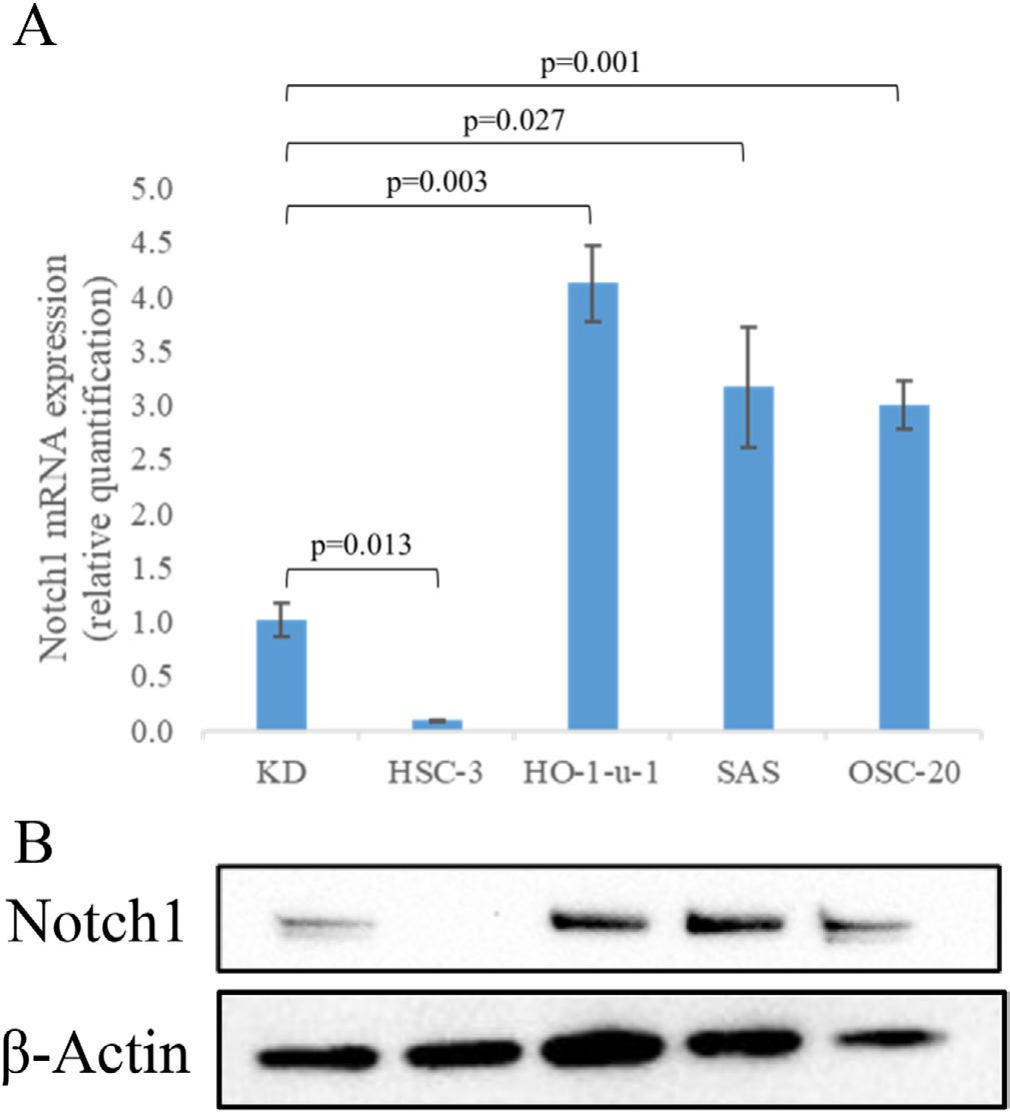
*Notch1* expression in the lip fibroblast cell line and oral squamous cell carcinoma (OSCC) cell lines. (A) Quantitative real‐time polymerase chain reaction analysis of *Notch1* expression. (B) Western blot analysis of Notch1 protein expression in OSCC cell lines. Abbreviation: KD, lip fibroblast cell line.

To confirm that the Notch signaling pathway plays a key role in OSCC tumorigenesis, the expression of *Notch1–4* was examined by qPCR in 16 cell lines, including fibroblasts of the liver cell line (KD). There were no significant differences among these cell lines in the expression of *Notch 2, 3*, and *4* (data not shown). Of the 15 OSCC cell lines, four were selected based on their *Notch1* expression profiles. We then measured *Notch1* expression levels in KD and HSC‐3, HO‐1‐u‐1, SAS, and OSC‐20 cells based on the standardized expression of GAPDH by qPCR (Figure 1A). We found that HO‐1‐u‐1, SAS, and OSC‐20 cells showed high expression of Notch1, while HSC‐3 cells showed lower expression of Notch1 than that of KD cells by WB (Figure 1B). Notch1 protein levels in HO‐1‐u‐1, SAS, and OSC‐20 cells were much higher than those in KD cells, and those in HSC‐3 cells were much lower than those in KD cells.

### 3.2 Notch1 Knockdown Led to Decreased Cell Proliferation, but Not Cell Migration

**FIGURE 2:**
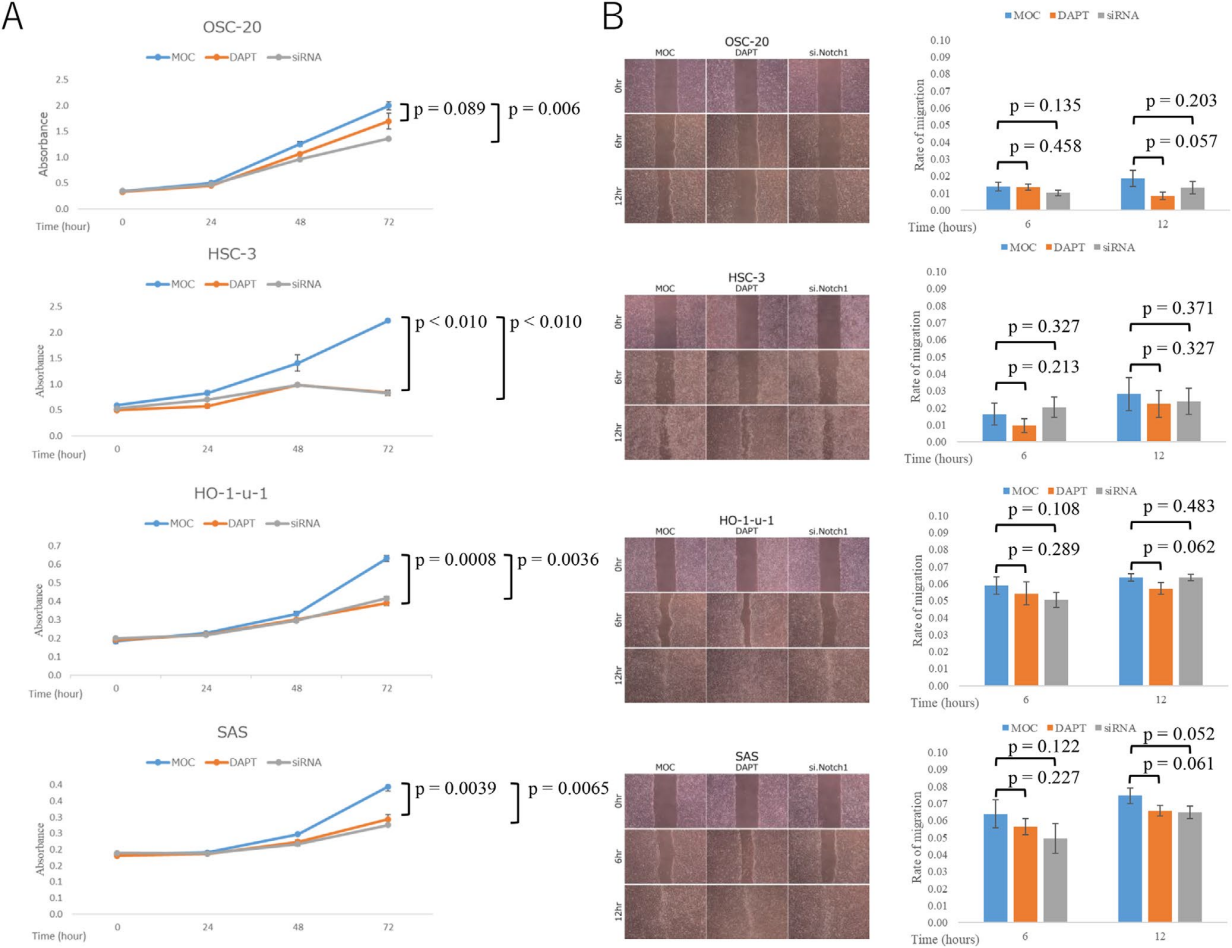
Effects of DAPT and *Notch1* knockdown on the biological behavior of oral squamous cell carcinoma (OSCC) cell lines. (A) Absorbance at 0, 24, 48, and 72 h after the addition of DAPT or after *Notch1* knockdown. Significant growth inhibition by DAPT was observed in HSC‐3, HO‐1‐u‐1, and SAS cell lines but not in OSC‐20 cells, *p < 0.05. (B) Microscopic images showing the rate of migration and wound closure at 6 and 12 h post‐wounding. There were no significant correlations between cell migration and DAPT in the four cell lines. KD.

To explore whether *Notch1* functions as a tumor suppressor or oncogene, we performed cell growth and cell invasion assays in the presence of Notch inhibition via DAPT treatment or siRNA‐mediated *Notch1* knockdown in OSCC cell lines. The results of the cell growth assay showed that DAPT treatment did not significantly suppress the growth of OSC‐20 cells with wild‐type *Notch1*, whereas it inhibited the growth of HSC‐3, HO‐1‐u‐1, and SAS cells with *Notch1* mutation after 72 h. However, siRNA‐mediated *Notch1* knockdown significantly suppressed the growth of OSC‐20, HSC‐3, HO‐1‐u‐1, and SAS cells irrespective of Notch1 status after 72 h (*p* < 0.05) (Figure 2A).

In contrast, in the wound healing assay, there was no significant difference between DAPT treatment and siRNA *Notch1* knockdown in the four cell lines at 6 and 12 h (Figure 2B).

### 3.3 Analysis of the Mutation Distribution Across Notch1 Functional Domains

We analyzed the mutation distribution in the Notch1 functional domains in OSCC cell lines using whole‐exome sequencing. Two mutations were located in the EGF‐like domain of the Notch1 extracellular region. One of the two, a mutation in exon 7, was commonly observed in SAS and HO‐1‐u‐1 cells. The other mutation was located in exon 13 of HO‐1‐u‐1. An amino acid sequence comparison of the mutation sites among species revealed that two *Notch1* missense mutations (G672V and E415K) were highly conserved among vertebrate *Notch1* orthologs, suggesting significant functional effects of these mutations in the pathogenesis of OSCC. However, a splice site mutation in exon 31 was located in the Notch1 intracellular region of HSC‐3 cells (Table 1).

**TABLE 1:** Mutational distribution of the exon functional domains of *Notch1*.

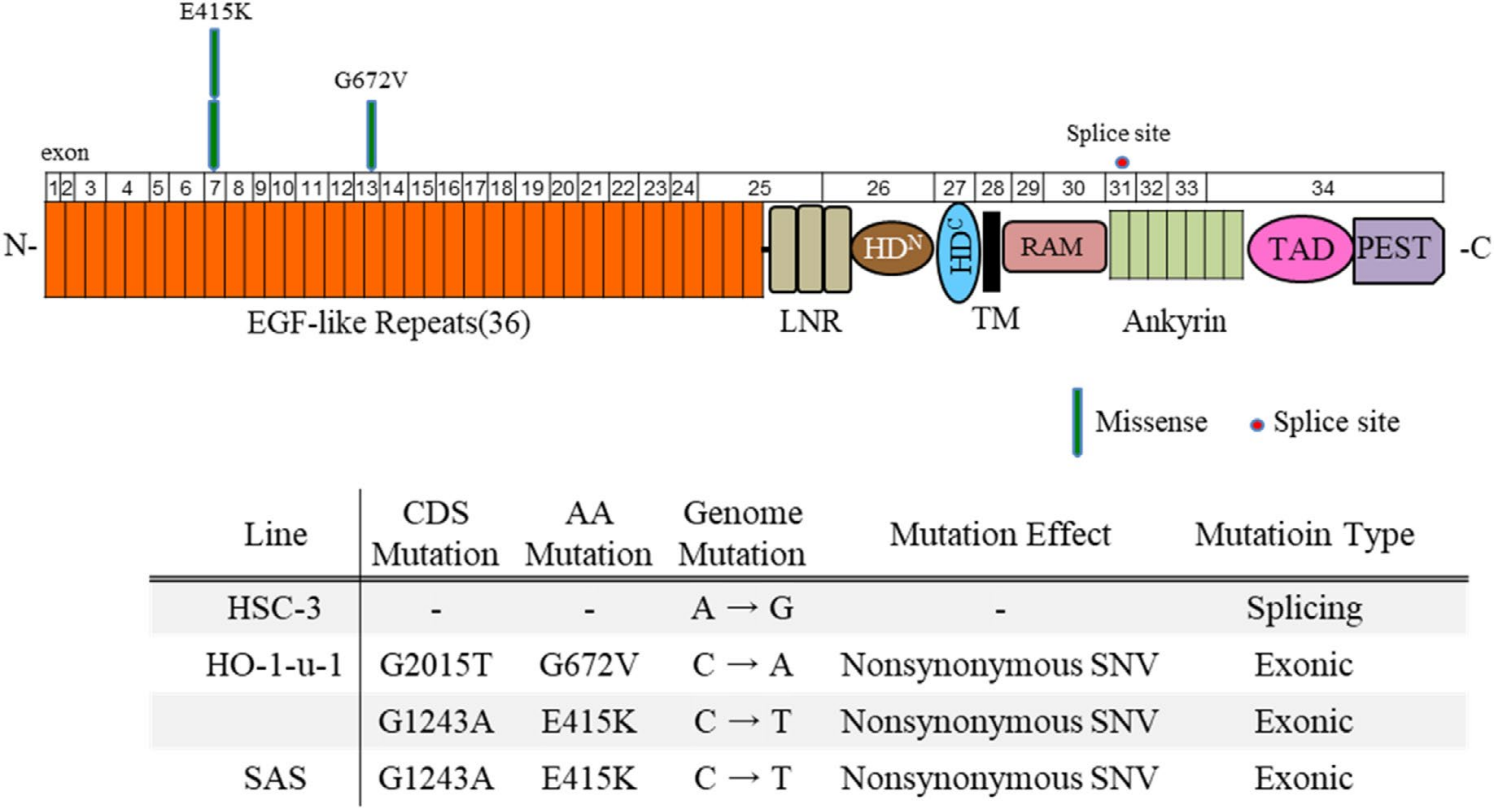

*Note:* Each arrowhead represents a nonsynonymous mutation in oral squamous cell carcinoma (OSCC) cell lines (HSC‐3, HO ‐ 1 ‐ u ‐ 1, and SAS). Individual exons are represented as numbered boxes. The conserved domains were mapped from UniProt. The green down‐pointing arrows indicate missense mutations, while circles indicate splice site mutations. EGF‐like, epidermal growth factor‐like; LNR, Lin12/Notch repeat; HD^N^, heterodimerisation–N terminal; HD^C^, heterodimerisation–C‐terminal; HD, heterodimerisation; TM, transmembrane; RAM, RBP‐associated molecule; Ankyrin, CDC10/ankyrin domain; TAD, transactivating domain; PEST, a region rich in proline (P), glutamate (E), serine (S) and threonine (T).

### 3.4 Alteration of PD‐L1 Protein Level by Notch Inhibitor or Notch1 Knockdown

**FIGURE 3:**
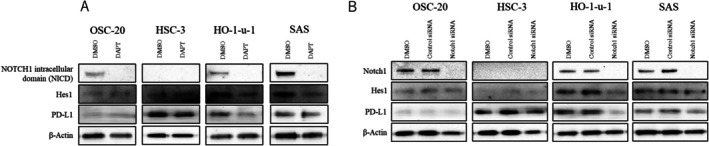
DAPT or *Notch1* knockdown regulates Hes1 and intrinsic programmed cell death ligand 1 (PD‐L1) expression in oral squamous cell carcinoma (OSCC). (A) The anti‐Notch1 intracellular domain (NICD) antibody recognizes the C‐terminal domain of Notch1. The expression levels of PD‐L1 and the Notch‐signaling target Hes1 remained stable in OSC‐20 and HSC‐3 cells when the Notch‐signaling pathway was suppressed by DAPT. However, the expression of PD‐L1 and Hes1 was down‐regulated in HO‐1‐u‐1 and SAS cells upon Notch‐signaling pathway suppression by DAPT. (B) Expression of PD‐L1 and Hes1 remained stable in OSC‐20 and HSC‐3 cells upon *Notch1* knockdown using siRNA. However, their expression was downregulated in HO‐1‐u‐1 and SAS cells upon *Notch1* knockdown.

We assessed whether DAPT treatment or Notch1 knockdown affected PD‐L1 expression. Notably, intrinsic PD‐L1 protein levels in OSCC depend on the cell line used. DAPT did not affect PD‐L1 expression in OSC‐20 cells with no mutation of *Notch1*. Additionally, DAPT did not affect the high expression of PD‐L1 in HSC‐3 cells with NICD mutation of *Notch1*. However, DAPT inhibited the expression of Hes1, a target gene of the Notch signaling pathway, and decreased the expression of PD‐L1 in HO‐1‐u‐1 cells but not in SAS cells, as shown by WB (Figure 3A).

We assessed whether Notch1 siRNA affects PD‐L1 expression. Notch1 siRNA did not affect PD‐L1 expression in OSC‐20 cells with no mutation of *Notch1*. Also, it did not affect the high expression of PD‐L1 or Hes1 in HSC‐3 cells. However, Notch1 siRNA inhibited the expression of Hes1 and decreased the expression of PD‐L1 in HO‐1‐u‐1 cells but not in SAS cells, as shown by WB (Figure 3B). Notch pathway inhibition enhances intracellular PD‐L1 expression in a Notch signaling‐dependent manner in OSC‐20, HSC‐3, and HO‐1‐u‐1 cells.

### 3.5 Alteration of PD‐L1 Expression by Flow Cytometry Analysis

**FIGURE 4:**
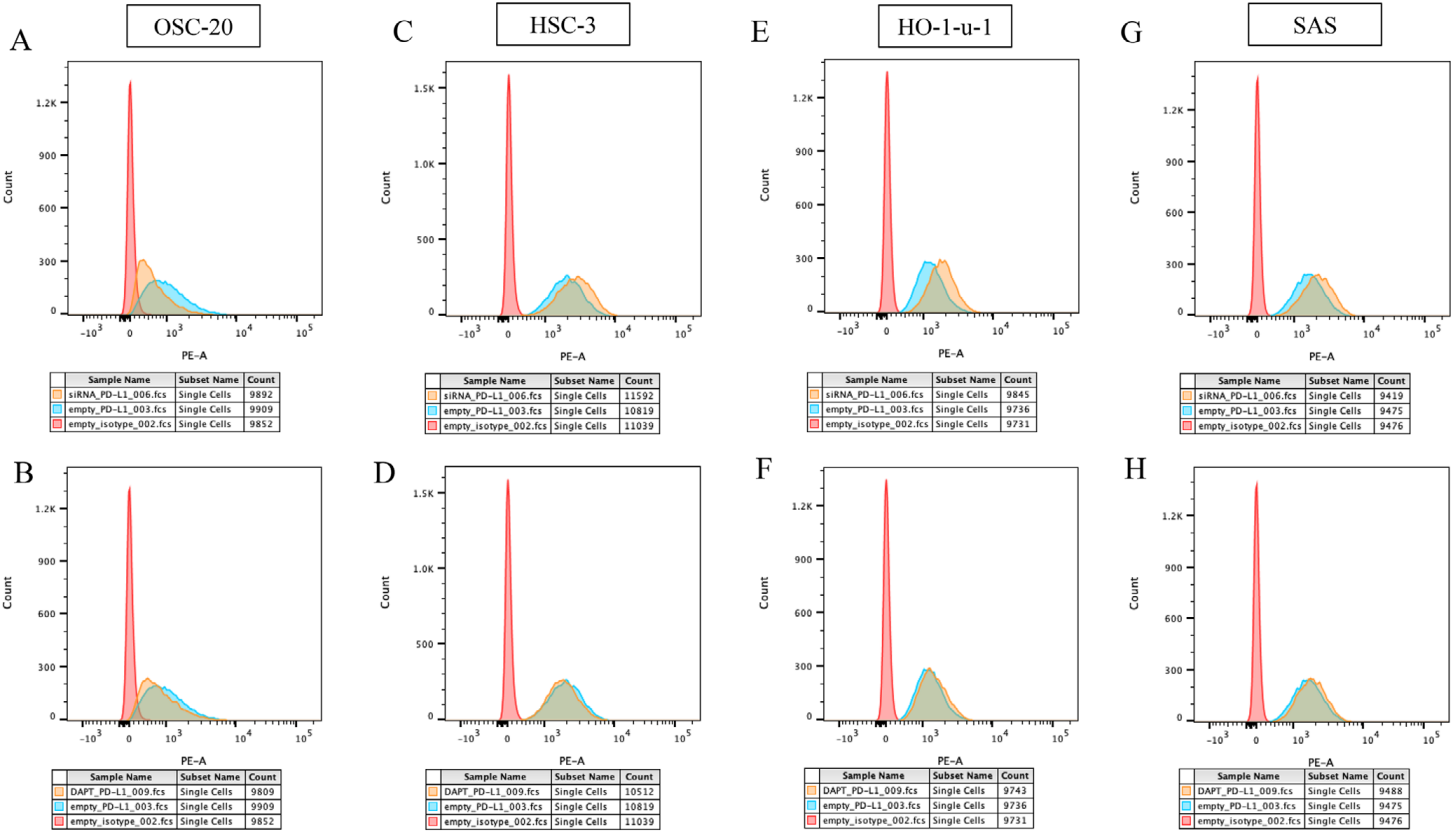
Flow cytometry histograms presenting the cell surface expression of PD‐L1. The histograms of OSC‐20 (A), HSC‐3 (C), HO‐1‐u‐1 (E), and SAS cells (G) were not changed following DAPT treatment. The histograms of OSC‐20 (B), HSC‐3 (D), HO‐1‐u‐1 (F), and SAS cells (H) were dynamically changed following *Notch1* knockdown.

The fluorescence count of PD‐L1–positive cells in OSC‐20 cells, which lacked the Notch1 mutation, remained unchanged by Notch1 knockdown compared to that of empty PD‐L1 (Figure 4A), and the fluorescence count of PD‐L1–positive cells was slightly decreased by DAPT treatment compared to that of empty PD‐L1 (Figure 4B). The fluorescence count of PD‐L1–positive cells in HSC‐3 cells carrying Notch1 mutations was highly increased by Notch1 knockdown (Figure 4C) and moderately decreased by DAPT treatment (Figure 4D). Additionally, the fluorescence count of PD‐L1–positive cells was slightly increased following Notch1 knockdown in HO‐1‐u‐1 cells (Figure 4E). The fluorescence counts of both HO‐1‐u‐1 and SAS cells carrying Notch1 mutations remained almost unchanged following DAPT treatment (Figure 4F,H).

However, the fluorescence count of PD‐L1–positive cells in SAS cells was almost unchanged following Notch1 knockdown (Figure 4G). Thus, PD‐L1–positive cell counts were slightly altered by DAPT treatment in OSC‐20 cells and by Notch1 knockdown in HO‐1‐U‐1 cells. In contrast, PD‐L1–positive cells were markedly altered by both Notch1 knockdown and DAPT treatment in HSC‐3 cells but showed almost no change following either treatment in SAS cells.

### 3.6 Clinical Parameter Analysis of *Notch 1* Mutation

**FIGURE 5:**
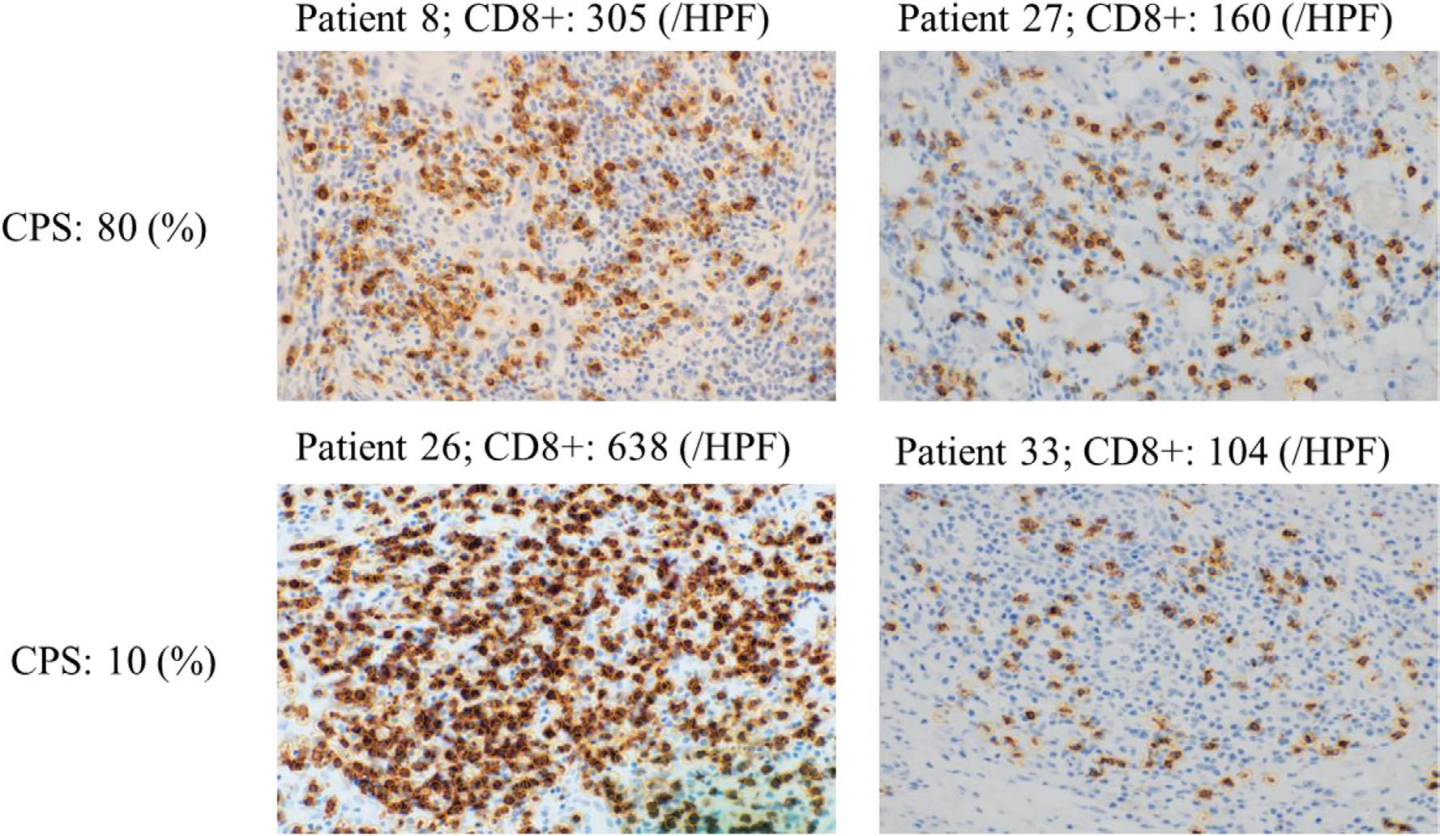
The image of tumor‐infiltrating CD8^+^ T cells (×400). The representative samples of low and high tumor‐infiltrating CD8^+^ T cell densities in patients with oral squamous cell carcinoma (OSCC). The immunohistochemical (IHC) image in the upper left is from case No. 8; the upper right is case No. 27; the lower left is case No. 26; and the lower right is case No. 33. The score of tumor‐infiltrating CD8^+^ T cells differs among Notch1 mutation groups with CPS > 1. Abbreviation: CPS, combined positive score.

To explore the role of *Notch1* mutation, we selected 12 patients with PD‐L1 expression > 1% from the 47 patients in our previous study and analyzed their clinicopathological parameters. However, no significant correlations were found between these parameters and *Notch1* mutations owing to the small cohort size, as shown in Table 2. We then assessed the correlation between Notch1 mutations and CD8^+^ T cell tumor infiltration. Five of seven patients (71%) with a *Notch1* mutation in our previous study were retrospectively examined by IHC staining for PD‐L1 and tumor‐infiltrating CD8^+^ T cells, as shown in Table 3. CD8^+^ T cell counts were stratified into < 200 and > 200, as the average CD8^+^ T cell count was 200. An image of CD8^+^ T cells infiltration by IHC is shown in Figure 5.

**TABLE 2:** Clinicopathological parameters of 12 patients with oral squamous cell carcinoma (OSCC) according to *Notch1* mutation.

Variables	Number of cases	NOTCH1 mutation	
		Negative	Positive
Age			
Median	67.5 years		
Range	57–84 years		
Gender			
Male	9 (75.0%)	5 (41.7%)	4 (33.3%)
Female	3 (25.0%)	0 (0.0%)	3 (25.0%)
Clinical T‐stage			
T1	4 (33.3%)	1 (8.3%)	3 (25.0%)
T2	6 (50.0%)	3 (25.0%)	3 (25.0%)
T3	0 (0.0%)	0 (0.0%)	0 (0.0%)
T4	2 (16.7%)	1 (8.3%)	1 (8.3%)
Clinical N‐stage			
N0	8 (66.7%)	3 (25.0%)	5 (41.7%)
N1	2 (16.7%)	1 (8.3%)	1 (8.3%)
N2	2 (16.7%)	1 (8.3%)	1 (8.3%)
Clinical stage			
I	2 (16.7%)	0 (0.0%)	2 (16.7%)
II	5 (41.7%)	2 (16.7%)	3 (25.0%)
III	2 (16.7%)	1 (8.3%)	1 (8.3%)
IV	3 (25.0%)	2 (16.7%)	1 (8.3%)
Lymph node metastasis			
Negative	8 (66.7%)	4 (33.3%)	4 (33.3%)
Positive	4 (33.3%)	1 (8.3%)	3 (25.0%)
Differentiation			
Well	6 (50.0%)	2 (16.7%)	4 (33.3%)
Moderate	6 (50.0%)	3 (25.0%)	3 (25.0%)
Poorly	0 (0.0%)	0 (0.0%)	0 (0.0%)

**TABLE 3:** Overview of clinicopathological parameters, somatic *Notch1* mutations, PD‐L1 protein expression by combined positive score (CPS), and tumor‐infiltrating CD8^+^ T cells in 12 patients with oral squamous cell carcinoma (OSCC).

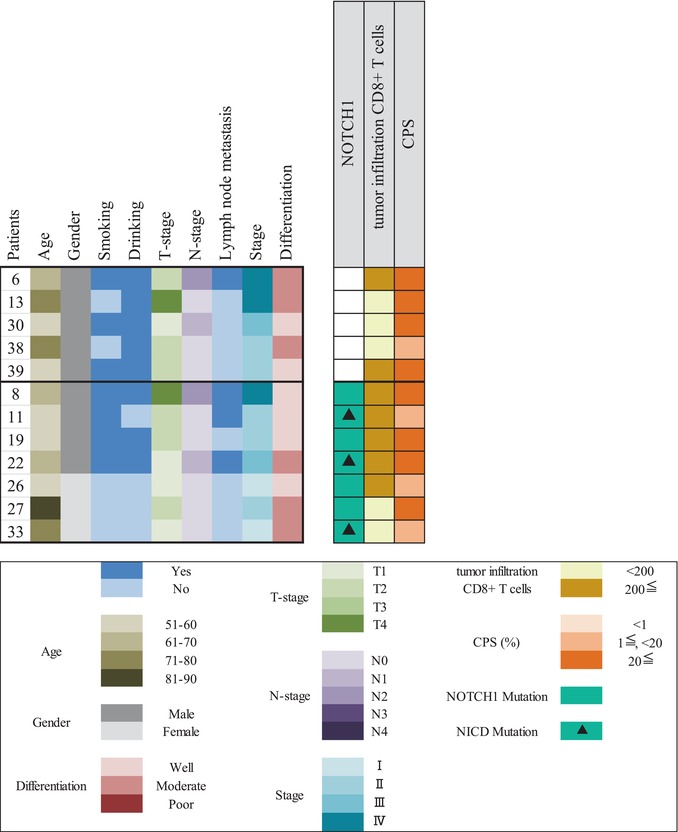

In the present study, five of the 12 patients did not have *Notch1* mutations, and two of the five patients had mutations in CPS > 20 and CD8^+^ T cell counts > 200, as shown in Table 3. Based on CD8^+^ T cell counts, > 200 was observed in two of five patients (40%) and < 200 in three of five patients (60%). Conversely, seven of the 12 patients had *Notch1* mutations. Three of the seven patients harbored a mutation in the *Notch1* NICD site; however, no significant correlation was found between CD8^+^ T cell count and CPS. CD8^+^ T cell counts > 200 were observed in five of seven patients (71.4%), and < 200 in two of seven patients (28.6%).

Overall, tumor infiltration by CD8^+^ cells was higher in the group with *Notch1* mutations than in the group without. This suggests a possible correlation between *Notch1* mutation with CPS > 1 and increased CD8^+^ T cell infiltration.

### 3.7 *Notch1* Mutations Were Associated With an Inflamed Microenvironment

**FIGURE 6:**
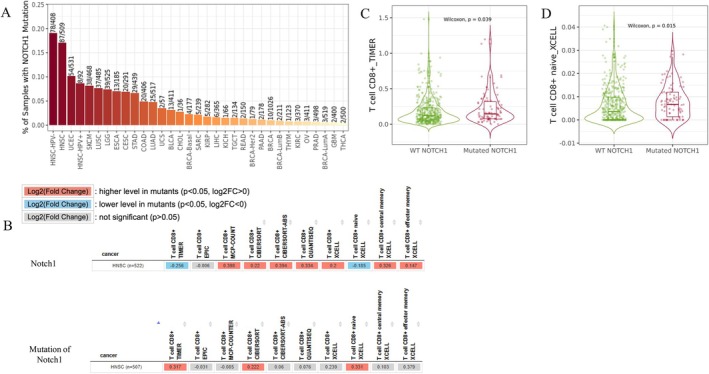
Profiles of *Notch1* mutation and immune infiltration of CD8^+^ T cells. Timer 2.0 showing the *Notch1* mutation frequency in pan‐cancer (A). Overall review of CD8^+^ T cells in WT vs. mutated *Notch1* by Spearman correlation analysis (B). Comparison of WT *Notch1* versus mutated *Notch1* in CD8^+^ T cells (C) and WT versus mutated *Notch1* in CD8^+^ naïve T cells (D).

We subsequently investigated whether *Notch1* mutations could lead to remodeling of the immune microenvironment in HNSCC. According to the TIMER2.0 website, HNSCC‐HPV (−), HNSCC, and HNSCC‐HPV (+) had the top five highest mutation frequencies in the samples (Figure 6A), and we further evaluated the association between *NOTCH1* mutation status and immune cell subtypes.

In HNSCC, mutated type (MT) *Notch1* showed a significant positive correlation with CD8^+^ T cell (TIMER), CD8^+^ T cell (CIBERSORT), and CD8^+^ naïve T cell infiltration levels (Figure 6B), while wild‐type *Notch1* (WT) showed a significant negative correlation with CD8^+^ T cell (TIMER) and CD8^+^ naïve T cell (XCELL). Notably, MT *Notch1* had significantly higher levels of CD8^+^ T cells than WT *Notch1*, as estimated by the TIMER algorithm (*p* = 0.039, Figure 6B) and CD8^+^ naïve T cells by the XCELL algorithm (*p* = 0.015, Figure 6D).

## 4 Discussion

*Notch1* mutations have been implicated in the progression of various cancer types, including breast cancer, leukemias, HNSCC, and squamous cancers of the skin, esophagus, cervix, and lung. Comprehensive genomic analyses of HNSCC have revealed a high rate of *Notch1* mutations, making it the second most frequently mutated gene after *TP53* [19]. Notch1 controls genes involved in early differentiation, leading to different phenotypic consequences depending on the cancer's genetic background [20]. To confirm *Notch1* expression levels in different OSCC cell lines (HSC‐3, SAS, HO‐1‐u‐1, and OSC‐20) and the lip fibroblast cell line, we observed that the three cell lines expressed high levels of Notch1 mRNA and protein compared to those expressed by the lip fibroblast cell line. We also conducted cell proliferation and wound healing assays using a γ‐secretase inhibitor (DAPT) or Notch1 knockdown. No significant effect was observed from DAPT inhibition on cell growth in only OSC‐20 cells. However, OSC‐20, HSC‐3, HO‐1‐u‐1, and SAS cells, irrespective of Notch1 status, showed significantly suppressed cell growth following Notch1 knockdown after 72 h (*p* < 0.05; Figure 2A). There were no correlations between Notch1 expression and cell invasion in these cells (Figure 2B). Based on these results, we conclude that *Notch1* mutation may have a tumor‐suppressive role in OSCC cell lines. In the present study, we aimed to elucidate not only Notch1 biological function but also its immunogenic function in OSCC.

Interestingly, it was recently reported that *Notch1* mutations correlate with a higher tumor mutation burden (TMB) in non‐small cell lung cancer (NSCLC) [21]. It was reported that tumors with *Notch1* mutations displayed significantly higher TMB compared to WT Notch1 tumors. Weng et al. identified frequent *Notch1* mutations in the heterodimerization domain and/or the C‐terminal PEST domain in T‐cell acute lymphoblastic leukemia, which are considered oncogenic drivers [22]. This suggests that alterations in DNA damage‐ and repair‐related genes resulting from *Notch1* mutations could account for the differential mutational load [21], and the heterogeneity observed in mutational signatures across various tumor subtypes sheds light on their origin, clinical evolution, and potential therapeutic sensitivities [23].

In contrast, Wei et al. reported a significant negative correlation between *Notch1* mutation status and PD‐L1 protein expression in small cell lung carcinoma (SCLC). Additionally, SCLC patients with positive Delta‐like 3 expression, which belongs to the Notch ligand family and had no mutation in *Notch1*, exhibited higher PD‐L1 expression and may be more likely to benefit from PD‐1/PD‐L1 ICI treatment [24].

The present study aimed to investigate the interaction between *Notch1* mutations and PD‐L1 expression, which may enhance anti‐tumor immunity. To substantiate this, we first examined the correlation between Notch1 mutations and PD‐L1 expression in vitro. Among the four cell lines investigated, only three harbored Notch1 mutations. First, OSC‐20 cells, which had no mutations, showed no significant changes in PD‐L1 expression in the cells or on the cell surface. HSC‐3 carries a mutation in the NICD, and the signaling pathway does not function after entering the nucleus. Conversely, SAS cells appeared to show no effect on PD‐L1 expression on the cell surface, which may be partly attributed to differences in the mutation sites between SAS and HO‐1‐U‐1, indicating the possibility that another site may have been the functional mutation site. Although Notch1 knockdown did not alter intracellular protein expression, inhibition of Notch1 increased PD‐L1 expression on the cell surface. A similar trend was observed in HO‐1‐U‐1 cells, although a definitive difference in PD‐L1 expression was observed. Based on these results, we conclude that a wild type *Notch1* phenotype does not affect PD‐L1 expression. This result may be partially explained by a correlation between *Notch1* mutation status and PD‐L1 protein expression levels in the OSCC tumor microenvironment.

Subsequently, we investigated the interaction between *Notch1* mutations and tumor infiltration of CD8+ T cells for immune recognition. Recently, Duan et al. highlighted the heterogeneity of Notch1‐associated tumor immune infiltration across pan‐cancers [25], and Wang et al. highlighted the pivotal role of Notch1 signaling in modulating the tumor immune microenvironment [26]. In previous studies, when the TME was classified into four categories based on the level of tumor‐infiltrating T cells and PD‐L1 expression, an inflamed TME—considered as a paradigm of adaptive resistance mediated by the PD‐1/PD‐L1 pathway—was characterized by increased PD‐L1 expression and T cell infiltration [27, 28].

We analyzed this interaction using clinical OSCC samples and online bioinformatics tools (TIMER2.0). In OSCC samples, we retrospectively examined IHC data with or without *Notch1* mutations in cases with high CPS using clinical samples of tumor‐infiltrating CD8^+^ T cells. We did not observe a significant correlation between *Notch1* mutation in CPS‐high PD‐L1 and tumor‐infiltrating CD8^+^ T cells. Notably, there may be a trend suggesting that *Notch1* mutation in CPS‐high PD‐L1 is associated with CD8^+^ T cell infiltration.

Next, we examined the interaction between *Notch1* mutations and immune cell infiltration using TIMER2.0. Interestingly, MT *Notch1* had significantly higher levels of CD8^+^ T cells than WT *Notch1*, according to the TIMER algorithm (*p* = 0.039) and the CD8^+^ T naïve XCELL algorithm (*p* = 0.015) in HNSCC. However, our findings regarding this correlation (increased CD8^+^ naïve cells in MT *Notch1*) is modest and correlative in nature.

As a timely contribution, Shangkun et al. recently reported molecular evidence explaining changes in the immune microenvironment following the Notch signaling pathway. They focused on Forkhead box M1 (FOXM1), which is linked to tumor cell cycle, drug resistance, and tumor immune escape, and reported that FOXM1 affected PD‐L1 levels through the Notch pathway, thereby promoting gastric cancer (GC) progression. They concluded that Notch inhibition led to PD‐L1 up‐regulation, attenuated the inhibitory impact of FOXM1 overexpression on CD8+ T‐cell activation, and enhanced CD8+ T‐cell cytotoxicity against GC cells. Additionally, they confirmed that all of these effects were significantly reversed after FOXM1 knockdown [29]. In the present study, we highlighted this recent finding that the Notch signaling pathway influences PD‐L1 and CD8^+^ T cells in the tumor immune microenvironment, which may provide insight into a similar mechanism associated with Notch1 mutation.

Due to the limited number of OSCC samples with *Notch1* mutations in our study, further investigation is required to perform functional assays (e.g., co‐culture with CD8^+^ T cells) to directly demonstrate the immunological consequences of these molecular changes, address inconsistencies across cell lines through mutational and pathway profiling, and expand clinical validation with a larger cohort to ensure statistical rigor and avoid overinterpretation of non‐significant trends. Moreover, our results do not align clearly with in vitro or IHC findings and should be interpreted with caution. We plan to address these limitations in future studies.

In conclusion, our findings indicate that *Notch1* mutation is a potential therapeutic target for enhancing immune recognition in oral squamous cell carcinoma.

## Author Contributions

All authors had full access to the data in the study and take responsibility for the integrity of the data and the accuracy of the data analysis. Investigation, data analysis, and writing of the original draft: T.I. Study design, data analysis, writing, review, and editing: K.O. Data curation and methodology: T.N. and T.S. Data curation: S.M., K.N., K.S., and S.S. Investigation, methodology, and supervision: Y.S. Conceptualization, supervision, writing, review, and editing: A.M. All the authors approved the final version of the manuscript.

## Ethics Statement

This study was approved by the Institutional Review Board of Sapporo Medical University (approval number 332‐3410).

## Consent

Written informed consent was obtained from all patients prior to their participation in the study.

## Conflicts of Interest

The authors declare no conflicts of interest.

## Data Availability

The data that support the findings of this study are available from the corresponding author upon reasonable request.
